# Between Education and Opinion-Making: Dialogue between Didactic/*Didaktik* Models from Science Education and Science Communication in the Times of a Pandemic

**DOI:** 10.1007/s11191-020-00156-0

**Published:** 2020-09-09

**Authors:** Erik C. Fooladi

**Affiliations:** Department of Science and Mathematics, Volda University College, Volda, Norway

## Abstract

The fields of science education and science communication are said to have developed as disparate fields of research and practice, operating based on somewhat different logics and premises about their audiences. As the two fields share many of the same goals, arguments have been made for a rapprochement between the two. Drawing inspiration from a historical debate between the scholars John Dewey and Walter Lippmann, the present article is a case-oriented theoretical contribution applying models from science education and science communication in relation to a current socio-scientific issue (SSI), the COVID-19 pandemic. The main question of interest is how selected didactic (*didaktik*) models from science education and science communication can contribute to shed light on the present situation of an ongoing pandemic specifically and socio-scientific issues in general. Three models are synthesised to give a new composite model that may help communicators and educators understand, discuss, and analyse complex socio-scientific issues. The model is subsequently applied on the apparently contradictory issue of Norwegian and Swedish governments’ very different responses to the pandemic, despite grounding their decisions on largely the same scientific evidence and advice. Contrast is made by comparison with another SSI, anthropogenic global warming (AGW). It is argued that the exchange and combination of didactic models from the two fields may open new spaces for cross-pollination and cross-fertilisation to the mutual benefit of both science education and science communication.

## Introduction

Although the fields of science education and science communication share many common goals, they have traditionally operated based on somewhat different logics and premises about their audiences (Feinstein 2015). While science education research and practice by and large engage with formalised teaching and learning, science communication cannot permit itself the luxury of an audience obliged by the regulations of organised classes, mandatory participation and so forth. While the educator is at liberty to test learning outcomes, the communicator is often left in the dark in terms of what the communicatee takes away from the interaction. Science education would discuss teaching and learning, whereas science communication would often refrain from using a term such as teaching and rather focus on, for example, promotion of engagement. The science communicator, meanwhile, enjoys a greater flexibility to focus on any topic of relevance, independent of the limitations of curriculum or subject content. The two fields operate under different social contracts, so to speak. However, while the actors of science education (broadly understood as educators and students) share an arena with its own social mechanisms and discourses, they are at the same time part of a broader public and thus actors on the playground of science communication. Taken together, this can be conceptualised as a division of labour between the two fields. Nevertheless, according to Baram-Tsabari and Osborne (2015, p. 135), they “have evolved as disparate academic fields where each pays little attention to the other”, and the two scholars consequently call for a rapprochement between the two fields.

In the present case-oriented theoretical contribution, I seek to explore intersections and modes of interplay between science education and science communication by invoking selected didactic^1^ models from the two and applying them to the current socio-scientific (SSI) issue of the ongoing COVID-19 pandemic. I contextualise this in a comparison of the two neighbouring countries Norway and Sweden. The text is deliberately written in the midst of the pandemic, not allowing the luxury of hindsight for myself, researchers of the pandemic, students/teachers or the public at large. The tentative nature of our situation, as scientific knowledge is accumulated in front of our very eyes day by day, is tangible in what has been termed by anthropologists a liminal phase/space, a rite of passage. A new reality is unfolding around us, and we expect to come out on the other side transformed, perhaps in some ways for the better (Göker 2020; Hylland Eriksen 2020).

**Fig. 1:**
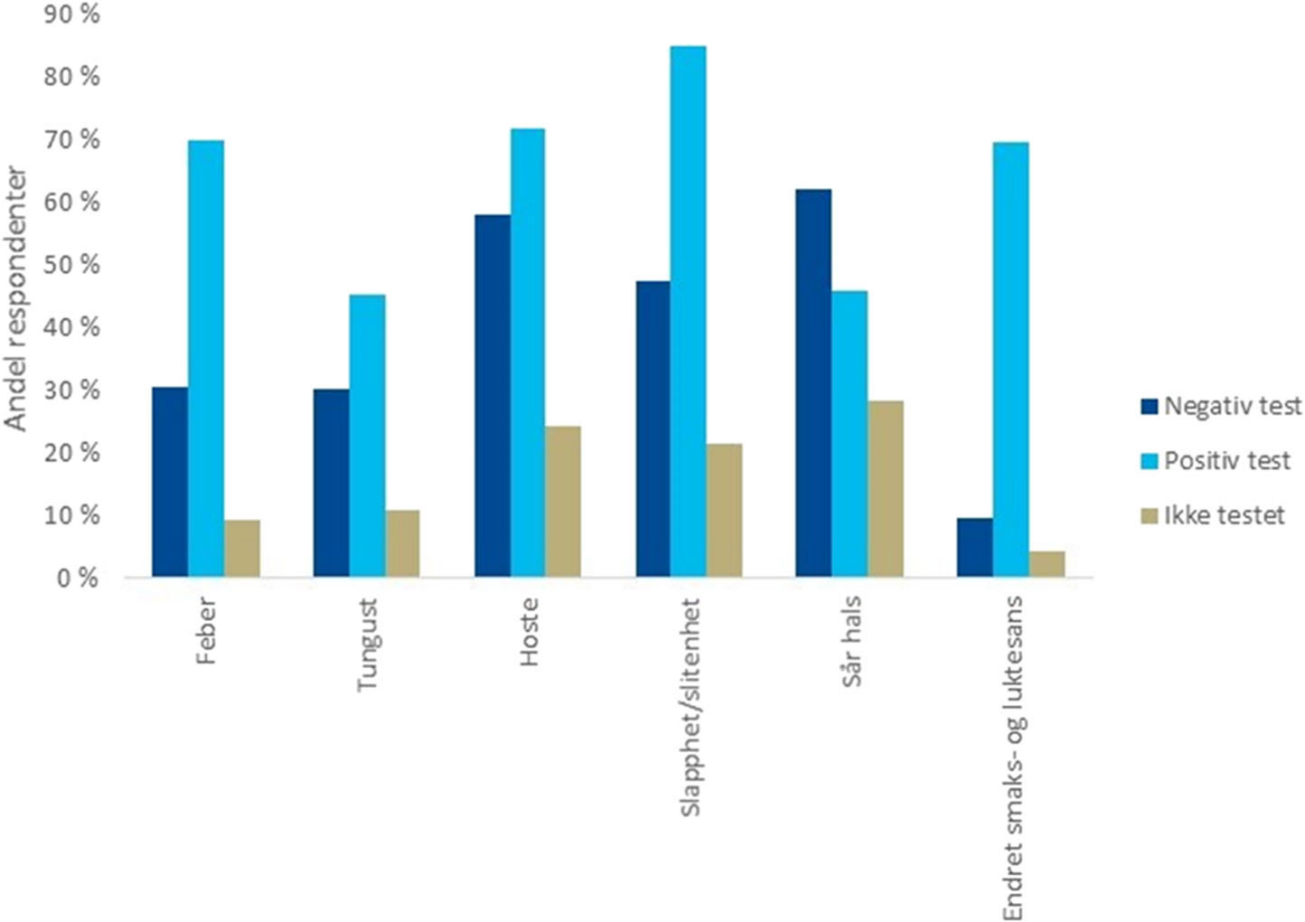
Diagram of initial findings from the Norwegian Corona study (Department of Microbiology, Oslo University Hospital 2020) distributed in an email newsletter to the survey respondents in May 2020. This is an illustrative example of researchers inviting respondents to understand or engage with their research. The diagram shows the proportion of respondents with various COVID-19 symptoms vs. infection status. Diagram used with permission

Living in the time of COVID-19 is not only a condition of observing others, but is about being personally affected regardless of whether one is infected. The media pressure also feels unprecedented, with the pandemic dominating virtually every newscast. We can follow the development on a day-to-day basis through online statistical services, interviews with leading researchers, and a scientific debate that appears to be wide open for everyone to follow. However, as citizens, we are not only *observing* scientists collecting data from labs and field studies to produce knowledge; we *are* the lab. Still, we are more than lab animals that passively provide data for science because we as citizens can choose to take an active role. We are even invited to participate—for example, by downloading tracking apps that feed data to research groups, or by contributing to national or international surveys distributed through social media. In some instances, the researchers may even return the favour by rewarding survey participants with updates on their most recent findings, how they have used the data to inform decision-making authorities and how far they have come in the process of publication in international research arenas (Fig. 1). A survey respondent may thus ask herself: “Am I now *subject to*, *an observer of, or a participant in* scientific research?”

Every day, we receive a continuous feed of new information through edited and social media. If we wish, we can engage ourselves in this public debate by, for example, joining social media groups such as ‘My child is not going to be a guinea pig for COVID-19’, a Norwegian Facebook group arguing against the (presumed premature) reopening of schools and kindergartens. Alternatively, we could use our voice or pen to argue against such groups by way of contributions to edited media (e.g. Vogt et al. 2020), or we could attend public demonstrations to show our support of or opposition to a certain matter. Rephrased in the language of science education research: we have the choice to be passive observers or active agents in an ongoing socio-scientific issue. The difference from many other SSIs taught in school is that this time we can both feed data into ongoing research and engage ourselves on a political level. We can engage in exploring *both* first-hand data mining and second-hand inquiry on an issue where researchers communicate both uncertainty and disagreement openly in the public sphere. Academic debate is out in the open in a, possibly, unprecedented manner. But are the majority of us, lay people rather than pandemic experts, sufficiently competent to expect to be heard? Do we deserve the right to opinion? What, then, about non-experts in leading positions forced to make decisions on local and national levels? In the spring of 2020, we could observe that governments in neighbouring and presumably similar countries, Norway and Sweden, chose starkly different ways to approach this pandemic, based on the very same research-based knowledge—Norway with lockdown, Sweden maintaining an open society. Consequently, questions of the right to opinion and epistemic power relations appear to be at the heart of decisions made on the personal level, communal/local levels and for those deciding on behalf of millions of others.^2^.

The present situation clearly has relevance to both science communication and science education. The two fields deal with many of the same issues and share many of the same goals (for a description of overlaps, similarities and differences between science education and communication, see Baram-Tsabari and Osborne 2015). As an example, students may discuss an SSI in science class at school (science education) and encounter the same topic in less formalised situations when reading a newspaper, watching a documentary at home or visiting a science museum (science communication). If the teacher uses the newspaper article, documentary or museum in her teaching, science education will be using science communication for purposes of formal education.

In my daily work with science education, at the same time being involved in science communication, I find that this fast-paced socio-scientific issue, still in its liminal phase, calls for ideas and thinking from both these fields. Albeit with mixed emotions due to the severity of the situation, it has opened a new space for cross-pollination and cross-fertilisation between the two. The approach chosen herein is to look for theoretical models from the two fields that may complement, or overlap, and thereby function as guiding didactic models (Zidny et al. 2020). Such models can help to clarify choices (why? what? how?), sorting out the elements of the landscape and thus promote a richer perspective on a given SSI. The main question of interest for the present inquiry is thus *how selected didactic models from science education and science communication can contribute to shed light on the present situation of an ongoing pandemic in particular, and socio-scientific issues in general, to the mutual benefit of both fields.*

In the following sections, I will situate this question in the case of the Norwegian versus Swedish response to the COVID-19 pandemic during spring and summer of 2020, thus setting the stage to put the theoretical models to the test in a concrete real-world situation. Comparison is also made with another SSI, anthropogenic global warming (AGW).

### The Case: Norway and Sweden, Neighbours but Still Very Different

Norway and Sweden can be considered well-functioning democracies with moderate political parties in governing positions, Norway with a Conservative-centre coalition government and Sweden ruled by a coalition between the Social Democratic and the Green Party. The political climate cannot be said to be dominated by either populist-or extreme wing parties nor by parties influenced by conspiracy theories or pronounced scepticism towards science-based knowledge. As seen from media reports, both governments appear to have a close and productive dialogue with their respective epidemiologic expertise, such as the Norwegian Institute of Public Health and the Public Health Agency of Sweden. Still, the two countries have chosen very different strategies towards COVID-19. Sweden has, at the time of writing this text (May–August 2020), Sweden has maintained an approach with few restrictions, and has done so from the beginning. Schools, kindergartens, restaurants and bars have been kept open. The government informs and requests the public to adhere to social/physical distancing and other protective measures without the application of public regulations such as forced shutdown. Norway, on the other hand, rapidly went into shutdown of schools, kindergartens, universities and most service industries and later slowly relieved the restrictions. Children and adolescents living along the border, only a few kilometres apart and perhaps being friends across the border, experienced very different everyday situations. A student on the Swedish side has gone to school every day, played football or attended music classes. The friend across the border has had to stay home, receiving teaching on the Internet, with football practice suspended and music classes at best given via online video teaching. Movement across the border is restricted, as Swedish areas are flagged by the Norwegian government as ‘red zones’. Businesses on the Swedish side are struggling as a result of the sudden absence of their main customer base of Norwegian shoppers (for some businesses, 95 percent of their sales are to customers travelling across the border). Still, both countries, or COVID-19 regimes, draw on the same body of science when making their decisions on local and national levels. Notably, the Swedish government has adhered *more* closely to the advice of the experts than the Norwegian, not less, as the political decision to close Norwegian schools and kindergartens, for example, was done for reasons other than on the basis of scientific advice. The Swedish practice, however, may stem from a more complex set of reasons than only that of epidemiology. In a recent newspaper debate article, two Swedish law professors provided the argument that a Swedish lockdown would be in conflict with three paragraphs of the Swedish constitution that secure full freedom of movement for all citizens, also valid during a state of emergency in time of peace (Jonung and Nergelius 2020). They conclude that the Swedish government therefore lacks the tool of general lockdown employed in many other countries. Furthermore, the constitution sets authorities such as the Public Health Agency of Sweden apart from the government, giving it a more independent (albeit not absolute) level of authority and responsibility during COVID-19, often personified by state epidemiologist, Anders Tegnell.^3^ We may thus ask: are there such things as right or wrong in the liminal phase of the pandemic?

### Scientific Debates Laid Bare

Inherent in responses to new epi-/pandemics is a high degree of uncertainty as to how to plan and respond to the situation effectively, especially in the absence of a functioning vaccine. As such, research-based response is a race against time, while the disease evolves in the population. Behavioural interventions (hand washing, lock-/shutdowns, face masks, etc.) are promoted to reduce or curb the spread, although the scientific basis for such may be inferential (Schuchat et al. 2011) or subject to ongoing research (testified by Web of Science or Google Scholar searches for keywords “face mask + COVID”). According to Cairns et al. (2013), prevention and control of communicable disease depend to a large extent on trust: “trust in advice providers is a significant predeterminant of compliance” (p. 1550). What, then, if advice providers, experts, appear to disagree amongst themselves? Due to the extreme media coverage today in terms of both quantity and the level of public scrutiny of ongoing research, pandemic-related science lies open for everyone to watch and follow. It is not that science has not been transparent before this, but the internal debates in the sciences appear to be followed much more closely in both non-edited and edited media, not to mention social media. Academic debate, plurality and disagreements on key issues are out in the open but differently than is seen in, for example, the AGW issue, where the vast majority of researchers have reached consensus on major aspects. The public conception of such consensus may diverge from the scientific, such as the widespread popular belief that scientists are still divided on AGW. In that case, a ‘consensus gap’ is identified between the research community and public perception (Cook et al. 2013, 2016; Oreskes 2018; Reusswig 2013; Tol 2016). Conversely, on the present pandemic, disagreement between experts with an apparently comparable right to opinion appears to a certain extent to be real. Furthermore, experts that would in a normal situation be accepted as having the right to opinion are now questioned by other experts as not being ‘the right kind of expert’. An example from the Nordic situation is the Norwegian Institute of Public Health who considered it relatively safe to maintain open schools and kindergartens because infection via children is considered a minor (or even negligible?) risk, whereas a researcher and medical practitioner in children’s diseases interviewed in a renowned newspaper finds it “very unlikely that children do not infect others” (Elnan and Belgaux 2020). Therefore, this appears not to be a question of a consensus gap between public and expertise, thus distinguishing the present pandemic from AGW.

If we interpret the present situation as ‘fast-paced liminal’, with an unknown outcome but expecting a relatively swift (re)solution, we may hypothesise that scientific debate and polyphony are not (yet) detrimental to the public’s trust in science-based, albeit tentative, knowledge. The immediate implications of (not) adhering to science-based advice in the context of the pandemic may also contribute is this direction, such as a spontaneous upsurge of infections as a result of not adhering to recommendations of social/physical distancing, use of facial masks, etc. Anthropogenic global warming, on the other hand, operates under a very different time scale (‘slow-paced liminal’), where our actions today are not visible tomorrow. Reopening schools therefore becomes a contentious matter where opposite choices can be made, both justified by appealing to valid expert knowledge. The public is offered the opportunity to take sides with experts with comparable expertise but with different opinions and arguments, even with the same framing of the issue (I revert to the notion of framing below).

## Between Opinion-Making and Educating for Civic Participation

“By the end of the 12th grade, students should have gained sufficient knowledge of the practices, crosscutting concepts, and core ideas of science and engineering to engage in public discussions on science-related issues, to be critical consumers of scientific information related to their everyday lives […]” (National Research Council 2012, p. 9).

“Is it realistic to expect ordinary, non-expert citizens to contribute to complex policy debates in an informed and creative manner? Lippmann’s answer was a resounding ‘no’.” (Feinstein 2015, p.145).

In his position paper ‘Education, communication, and science in the public sphere’, Feinstein (2015) describes similarities and distinctions between science education and science communication. Based on a debate between John Dewey and Walter Lippmann in the 1920’s USA, he lays out two alternative lines of thinking that appear to have subsequently distinguished the perspectives of science education and science communication communities. A main difference in thinking between the two fields was/is the belief in whether non-expert citizens are equipped to contribute to complex science-related policy debates in an informed and creative manner. At the heart of the debate, writes Feinstein, is the question of citizens versus expertise. Put simply, the ‘Lippmann line’ is that the average citizen is generally not equipped to make informed decisions about societal issues where science plays a key role (in today’s science education language: socio-scientific issues). This is not to imply that the average citizen is unintelligent, but basically that the science underlying most issues of public interest is too complex, at a level far beyond what is attainable to teach in school, a matter which har been discussed also in contemporary science education (Norris 1995; Roberts and Gott 2010; Ryder 2001; Sadler 2004, 2009). A main question is whether we should aim at “enab[ling] people to understand the role of evidence in science [so] they can engage with and challenge the science that impacts on their lives” (Roberts and Gott 2010, p. 204). That would resonate with the National Research Council (NRC) framework quoted above, but is according to Lippmann overly optimistic on behalf of the learner. The takeaway message from Lippmann is that we can safely abandon any attempt at educating students towards scientifically informed civic engagement and instead seek to teach for trust-building in the expertise. A stronger version of this would be teaching and communicating for opinion-making. The previously mentioned example of communication for the prevention and control of communicable disease as described by Cairns et al. (2013) may be seen as lying along this line, seeking to build trust in advice providers (experts) and promote public compliance with expert recommendations. A milder approach was presented by (Ryder 2001, p. 34), stating that “[c]ases in which non-scientists become centrally involved in the design and interpretation of scientific research […] are likely to be extremely rare […], the main mechanism of engagement for the individuals [could be] that of asking meaningful questions”.

Dewey’s response to Lippmann was, not surprisingly, more optimistic on behalf of the public. His main critique of Lippmann was indeed of his notion of ‘the public’. Dewey agreed that the science underlying public decisions is often too complex to include in school curricula for all. However, in his perspective, ‘the public’ was not merely a collection of isolated individuals but rather consisted of groups of individuals with different capacities in mutual and synergistic social interaction. The takeaway message from Dewey is that the public as collective can surpass what is attainable for the sum of the individuals. The public consequently also has a greater right to opinion than that promoted by Lippmann.

### Is There Merit in the Dewey and Lippman Positions Today?

The reality of Lippman and Dewey’s USA was very different from today’s world with a higher level of education in the public as whole (Roser and Ortiz-Ospina 2016), better access to information and vastly increased flow of communication. Albeit still unevenly distributed geographically and culturally, social participation and equality have also made considerable leaps. Since the days of Dewey and Lippmann, we have seen examples of social and socio-scientific issues that have engaged the general population on a large scale in new ways (AGW, *Silent Spring*, HIV pandemic, gender equality and recent philosophical trends and perspectives such as postmodernism).^4^ We may thus ask if the layperson of today is better equipped to make knowledge-based decisions, as implied by the quote from the NRC above, compared with the contemporaries of Dewey and Lippmann. Reaching for more recent examples from education and communication research, there does not appear to be strong support for such a claim. At the brink of the Internet revolution, Norris (1995), drawing on Code, Harré and Hardwig, went so far as to say that most of what non-scientists can hope to achieve of scientific knowledge is based on trust rather than actual understanding. This *intellectual communalism*, he builds on the notion of *epistemic dependence* which is an unavoidable trait of knowledge acquisition. This is not to say that non-scientists do not have the ability or possibility to be intellectually independent, but that such independence is embedded in judgements of trust rather than one’s own full understanding of a phenomenon. Such intellectual communalism is also a central trait of communities of scientists/experts, who are invariably reliant on trusting each other. Hence, judgement of science-based claims is for non-scientists virtually always, and for scientists often, a question of *choosing which expert/authority to trust* rather than judging the veracity of the claim itself. A similar conclusion was reached by Bingle and Gaskell (1994), who described evaluation of knowledge claims as based on positivist versus social constructivist views of scientific knowledge. We can thus say that Norris’ (1995) account takes up the issue of trust as illustrated by Cairns et al. (2013) above, although the word ‘compliance’ used by the latter authors may lead one’s thoughts towards opinion-making rather than trust-building. This can perhaps stem from an absence of the deeper theoretical foundations of epistemic dependence described by Norris in the work of Cairns et al. (2013). A reliance on trust does not necessarily mean that non-experts are stripped of their rights. On the contrary, in cases where the expert knowledge either directly concerns or indirectly impinges on the lives of non-experts, they have a legitimate right to make judgements of experts, because the knowledge is extrapolated to domains outside the experts’ areas of expertise (Norris 1997). That is, there is a difference between evaluating scientific evidence per se and evaluating the role of a scientist in a decision-making process. The layperson has a larger say in the latter than in the former.

In the science education literature, Dewey’s notions of learning and teaching still stand strong. Intellectual communalism and epistemic dependence, or *inter*dependence (Norris 1995), appear already to have been anticipated by Dewey’s notion of ‘the public’, characterised by its knowledge-sharing social interactions. In our time of the Internet, a new trait has appeared through the vast increase of available information to the general public. With this follows an increased need for the individual to make credibility judgements of both the source and the content itself (e.g. Bråten et al. 2011). New arenas for people to create echo chambers, epistemic bubbles and so forth also appear. In this light, Dewey’s notion may appear overly optimistic as ‘the public’ may collectively misguide just as much as guide themselves.^5^ Anecdotal evidence in favour of Dewey’s position, on the other hand, is the vastly increased availability of information online and the advent of self-taught expert bloggers and online special interest communities that are able to relate to complex and advanced information and use this to build and share new knowledge.^6^ Although we now live in a quite different world, a century later, it appears legitimate to state that the two positions are alive and thriving and that the Lippmann-Dewey debate is still relevant in our dealings with socio-scientific issues.

## Models from Science Education and Science Communication on the Interaction Between Science and Society

Science education and science communication both provide theoretical models that can help understand the issues described above on a general/meta-level. A litmus test for theoretical/didactic models is the degree to which they are functional when applied in real-word situations, either to understand the situation itself (here, the pandemic) or as heuristics for informed action (e.g. rationales for teaching and/or communication). I here present selected models from the two fields which I believe supplement each other and thus provide a productive cross-pollination/cross-fertilisation.

### Models from Science Communication

**Fig. 2:**
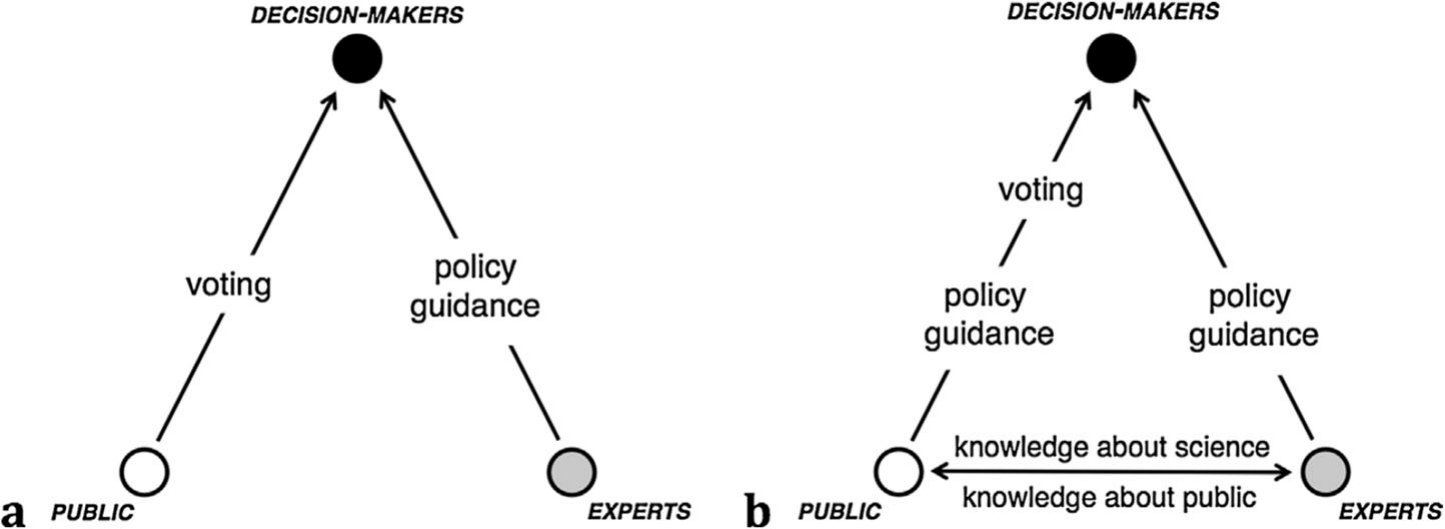
Representation of (**a**) Lippmann’s vision and (**b**) Dewey’s vision of the relationship between experts, the public and decision-makers (Feinstein 2015, p. 158). Figure used with permission

The range provided to us between the views of Lippmann and Dewey implies not only the question of citizens’ trust in science/experts but also a distinction in the degree of trust in the public by communicators, researchers and decision-makers. Feinstein (2015) illustrates Lippmann and Dewey’s positions on the relationship between experts and non-experts graphically as shown in Fig. 2.

**Fig. 3:**
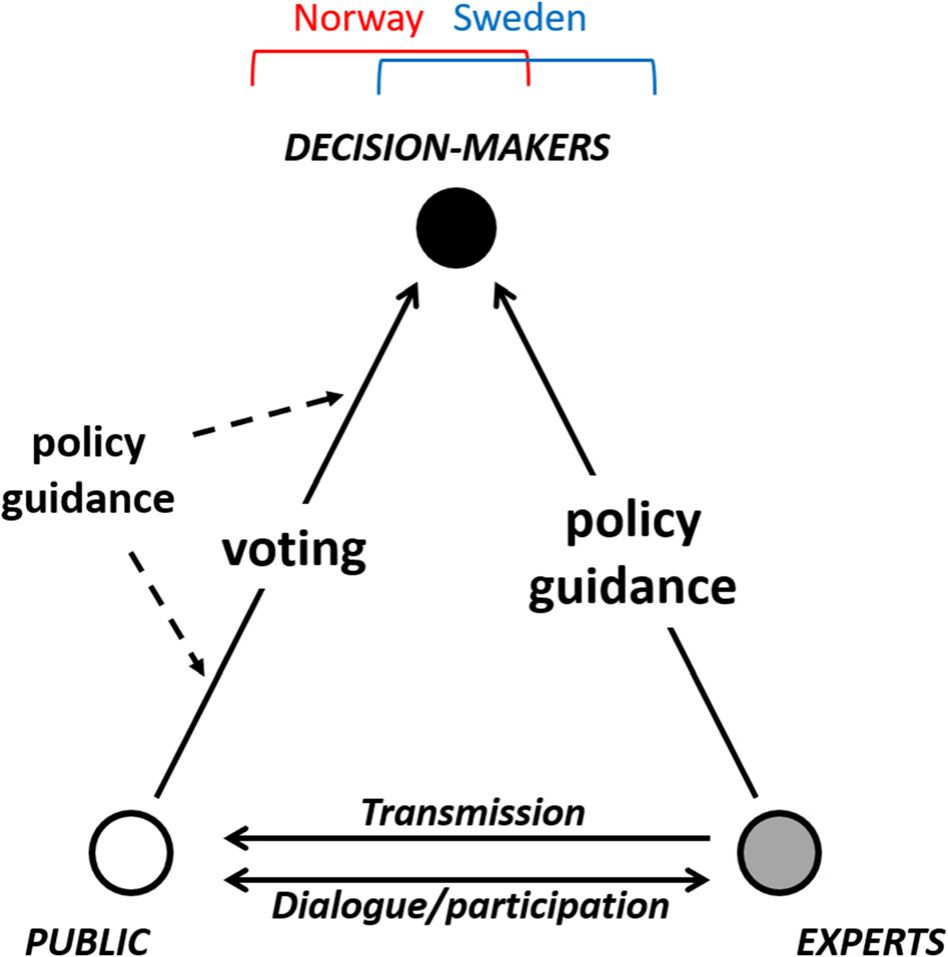
Revised version of Feinstein’s (2015) model of the relationship between experts, public and decision-makers applied to the Norwegian and Swedish situation during the COVID-19 pandemic. The bottom arrows indicate the direction of communication in various modes of communication, as described in Table 1

We may thus apply this model to the Norwegian versus Swedish pandemic situation, noting that despite the Norwegian Institute of Public Health *not* advocating the closing of schools, kindergartens and restaurants, due to low risk of infection amongst children, politicians *still* decided on lockdown. The Swedish government chose to keep schools, kindergartens and restaurants/bars open, based on the same scientific information. Applying the model in Fig. 2 in the present pandemic could look as indicated in Fig. 3, where the Norwegian situation leans towards the left-hand side and the Swedish towards the right-hand side of the triangle. The model is slightly altered compared with Fig. 2 to account for the constant pressure, or guidance, on decision-makers via social media and public debate in today’s societies.

Lippmann’s thinking can be said to have much in common with what has been termed the deficit model of the public in relation to science and scientific literacy (Baram-Tsabari and Osborne 2015; Bucchi 2008). The consequence of conceptualising the expert as ‘the knower’ and the public as the ‘not-knower’ is that communication must necessarily go from the former to the latter, a one-way, diffusionist, notion of communication between expertise and public. In this perspective, science communication is characterised by transmission, and ‘knowledge transfer’ becomes an idiomatic term. Quality communication is conceptualised as striving for the best possible way to ‘deliver the message’, hoping that the recipient (public) receives and understands the message and perhaps complies. As described by Bucchi (2008), this transmission notion of communication is later complemented by alternative modes of communication, notably those of dialogue, and participatory knowledge production (Table 1).

**Table 1:** Framework for science communication from Trench (2006) cited by Bucchi (2008, p. 69)

Communication model	Emphasis	Dominant versions in science communication	Aims	Ideological context
Transfer Popularisation One-way, one-time	Content	Deficit	Transferring knowledge	Scientism Technocracy Rhetoric of the knowledge economy
Consultation Negotiation Two-way, iterative	Context	Dialogue	Discussing implications of research	Social responsibility Culture
Knowledge co-production, deviations Multi-directional, open-ended	Content and context	Participation	Setting the aims, shaping the agenda of research	Civic science Democracy

In the dialogic, or transactional, mode of communication, the public is conceived as a competent partner in dialogue, not necessarily based on their factual scientific knowledge, but as legitimate actors through their part in the issue. Policies for science in society have recommended a shift towards more dialogic and transactional notions of communication (Siune et al. 2009). This resembles Ryder’s (2001) description of the mechanism for public engagement described above—that of asking meaningful questions, while at the same time not being in conflict with trust-based knowledge development, as described by Norris (1995, 1997). In the third mode, the participatory notion of communication, the public or laypersons are conceived as partners with the experts, actively engaging in the co-production of knowledge, citizen science projects being an example. In this light, the communication by researchers in the Norwegian corona virus study (Fig. 1) has a flavour of both dialogue and participatory modes of communication. As a respondent in the study, one may perceive oneself to be a participant in something larger, especially when the researchers actively keep the respondents in the loop by sharing updates with them. Seeking more dialogic and participatory models of teaching has likewise been a long-standing topic in educational research and practice. In this respect, science communication and science education appear to have a common interest in exploring more transactional modes of teaching and communication.

Reverting to the revised version of Feinstein’s (2015) model (Fig. 3), the transmission/diffusionist notion implies that the bottom horizontal arrow is unidirectional from expert to public, whereas the dialogic and participatory notions of communication imply that the arrow is bidirectional. Researchers may be influenced by public debate, and it is possible to envisage that they find some of their research questions in the public discourse, thereby implying an indirect participatory model. The public thus contributes to shaping the research agenda even without explicit and active participation (the present contribution is indeed a sign that it has) and thus may not perceive the dialogic aspect of the communication although the researcher may do so.

### A Model from Science Education Connecting Science, Argumentation and Decision-Making in Society

**Fig. 4:**
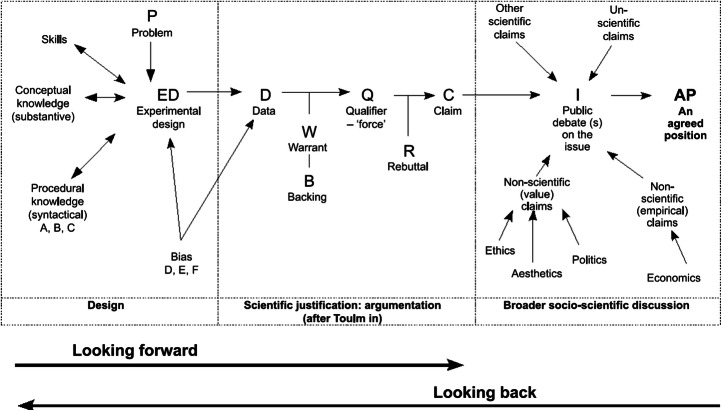
Model of interactions between science, argumentation and decision-making in the public sphere (Roberts and Gott 2010, p. 207). Figure used with permission

Roberts and Gott (2010) presented a descriptive model of interactions between science (teaching and/or research), argumentation and decision-making in the public sphere (Fig. 4). My choice of this model does not imply that other models from science education (see, for example, Kolstø 2001; Sadler et al. 2017; Zeidler et al. 2005) cannot productively be coupled with science communication models. The selected model, however, provides a succinct, visual and graphic overview of science and scientific practices on the one hand and social decision-making on the other, often also involving non-scientific factors (the right hand section of the figure has a clear resemblance to one found in Aikenhead 1985 but in addition aligns it with argumentation and with science content and practices). The model provides both detail, example and openness/flexibility, allowing it to be malleable to different SSIs and adapted to suit various educational levels (on the value of example and the particular vs. the general, see Meskin and Shapiro 2014). It does not provide an explicit structure or advice for the enactment of teaching, for which educators may draw on other models (e.g. Kolstø 2001; Sadler et al. 2017; Saunders and Rennie 2013; Zeidler et al. 2005).

The model comprises three main sections: scientific practices and knowledge production (including teaching) on the left-hand side give an overview of various components in scientific inquiry and knowledge-building to produce science-based information/data. The right-hand side visualises components that go into debates and social decision-making in the public sphere, drawing on science-derived knowledge as well as non-scientific factors such as ethical, aesthetic, political, economic and psychological ones. Unscientific claims (e.g. quasi-science) also play a role in debates and decision-making, be it on a private, local or societal level, and are therefore included here. The bridge between the data and knowledge claims used in decision-making is by way of argumentation, as indicated by Toulmin’s argumentation pattern (TAP) (Toulmin 1958/2003) making up the middle section (Toulmin’s argumentation pattern is expectedly well known in the science education community, e.g. Erduran and Jiménez-Aleixandre 2007; Osborne 2010). The model can be read in both directions (left–right; right–left). From the perspective of the researcher or citizen/learner doing first- or second-hand inquiry, the movement is from left to right, described as ‘looking forward’. The perspective of the public, learner or citizen, would normally be ‘looking back’, tracing the evidence used in science-based advice or decision-making back to its roots in evidence and practice. The actual situation is obviously more complex, as any piece of evidence (in Toulmin’s terms, ‘data’) can be used in an argumentative deliberation, be it scientific, non-scientific or unscientific. One might say that argumentation goes on everywhere and at all times, also within the left-hand and right-hand boxes, with many TAPs being in action at any one time.

What this model brings to the table is a visual and mental map that may assist in sorting out and understanding a socio-scientific issue. The public issue could be individuals’ everyday decisions: ‘do we run the risk sending our child to school when it opens?’ (parents); ‘is it safe for me to play with my friends?’ (child); ‘should I go to the pub?’ or ‘can I afford staying home from work?’ (individual citizen). It could be on a communal level, ‘should we open our school as recommended by the government or should we resist?’ (school leaders/councils; or national: ‘should we reopen the schools/pubs/borders?’ (government, public leaders). As science educators and communicators, we like to say that science is important for decision-making in society. However, as made visual in this model, scientific claims are only one factor in political or everyday decisions. Still, we would usually contend that decisions must rely strongly on scientific advice or at least rest on a foundation of scientific knowledge. The model can thus be used to shed light on the relative weight put on various aspects of a case, such as exposing bases for value judgements in today’s Norway and Sweden. On a governmental level, national health expert advice is basically the same in the two countries—that is to say, in Fig. 4, the left and middle boxes feed in the same science-based claim to the decision-makers. However, the agreed positions on the right-hand side in the two countries are radically different; hence the decisive factors are more than only scientific. In cases where scientific consensus is not clear, the situation is further complicated by the plurality of voices of the experts as previously described. Multiple and apparently contradictory scientific claims from the left-hand side must be balanced against each other, for subsequently to be weighed against non-scientific and unscientific ones.

## Drawing the Models Together to Apply to a Socio-Scientific Issue

In science education, socio-scientific issues are characterised by questions of a wicked nature, with no immediate or unambiguous answer (Sadler 2009). Often, they end up with appeal to authority argumentation strategies based on second-hand inquiries, particularly for the scientific knowledge claims of the debate on the right-hand side of Fig. 4. This is for several reasons, the first of which has already been discussed, namely that of the complexity and advanced nature of the underlying science, as pointed out by Norris and others. A second reason is that of temporality. The long-term nature of many SSIs dictates that the learners are not likely to see the effects of the topic of debate by the end of the teaching sequence, during their time at school, or even before they become parents or grandparents themselves. In short, the outcome of the conclusions drawn is not available within a reasonable time frame. Thirdly, many SSIs are not well suited for first-hand inquiry where students gather evidence themselves to produce data, warrants and claims to feed into a discussion. Students cannot experiment with their own health in a nutrition-related SSI, whilst genetically modified organisms (GMO), climate change and wildlife preservation are usually too complex for students to engage in first-hand explorations^7^ (see however, for example Fooladi (2013, 2020), Herranen et al. (2019) and Mata (2013) for some exceptions that still fall within Sadler’s (2009) definition of SSIs).

In the present pandemic, we are in the middle of an ‘experiment’ with data and knowledge appearing as we carry on with our lives. It goes without saying that it would be unethical, with potentially catastrophic results, to undertake first-hand experimentation with COVID-19 infection in school or by citizens of the public (the latter has been suggested, however, see Chappell and Singer (2020) and subsequent replies/debate).^8^ However, we do have access to data reported on a day-to-day basis through online services such as worldometers.info. This allows for the discussion of decision-making linked with development of the pandemic in real time. The model by Roberts and Gott (2010) can be used when exploring pandemic statistics (i.e. data in the middle box), using them in debates around which factors such as the economy, physical and mental health or juridical aspects, should weigh most when making decisions on school reopening, lifting a general lockdown, mandatory use of face masks, etc. Note that the right-hand box needs not be static but may function as a mind map where factors can be taken in or out. For instance, Fig. 4 does not specify juridical factors, although these would be amongst the non-scientific claims. In our present SSI context, this could be included explicitly.^9^ The statistical data available online give the SSI of an ongoing pandemic the potential for a larger first-hand inquiry component through data mining compared with other common SSIs used in teaching. It is expected that soon the scientific community will have reached a point where there is a basis for a greater degree of consensus. The scientific voices will be more unanimous, and the SSI of the COVID-19 pandemic will resemble other SSIs with a larger second-hand inquiry component and thus a stronger degree of appeal to authority argumentation. At that point however, it would still be possible to go back in time and emulate today’s situation where we yet do not have the luxury of hindsight—e.g. through a storyline methodology.

A takeaway message for science education from science communication could be to draw on the systematic and explicit manner in which this field conceptualises the different modes of communication, coupled with a meta-perspective on the relation between experts, the public, and the knowledge transactions in between (Fig. 2 and Fig. 3 and Table 1). Here, it is not necessarily the case that the modes of communication―transmission, dialogue, participation―are to be conceived as lying along a normative scale. Transmission is not always the least desirable nor participation the most desirable mode. This will depend on the context and content of the communication. Nor should this be confused with student-active approaches to teaching and learning. Rather, as a didactic model, it can be used to identify where one is epistemically situated when dealing with a certain SSI. Coupling the science communication model of Feinstein (2015) with Roberts and Gott’s (2010) model (Fig. 2 and Fig. 3 vs. Fig. 4), may help us to see more clearly the roles we can give scientific versus non-scientific knowledge claims. On this basis, we can make thoughtful decisions as educators and communicators on which notion of communication (Table 1), notably the role of science vs. learner/public, we find most suitable for a given situation, content or problem.

Feinstein reminds us that we in science education may have “created a substantial blind spot in our approach to public engagement with science—a blind spot that Lippmann would recognize instantly. [Namely, that] we assume that people who encounter science think of it in terms of science—that they interpret a news story on climate change through the lens of scientific evidence, or that their decision to protest Genetically Modified Organisms is grounded in an understanding of genetics” (Feinstein 2015, p.151). The public’s failure to coordinate their choices according to scientific knowledge is thus interpreted as failing to understand the science, and a better understanding of the science would result in a different, more desirable, choice. As shown in Roberts and Gott’s (2010) diagram, this may not be the case at all. Rather, the choice could have been made based on a deliberation where different aspects are weighed against each other and where the science is found to be of less weight in the final judgement. Feinstein invites us to be aware of whether we as science educators too readily conceptualise the right-hand box in Fig. 4 as mainly a product of the left-hand box. The Norway vs. Sweden example exposes this, pointing to different value judgements, or weight, given to what should be the decisive information when enforcing shutdowns/lockdown or not. Feinstein draws on the notion of *framing* from the field of communication, as a viable tool for the educator and educational researcher. Feinstein’s critique may thus be rephrased as a reminder to be conscious of *how an SSI is framed* when included in teaching. In science education, framing a story as science-based comes naturally (even automatically), whereas in the world of communication and media, such a framing may be considered painting a skewed picture as other factors are suppressed or disregarded (Fig. 4, right-hand box). Roberts and Gott’s model is however a didactic tool that allows for inclusion of this without necessarily introducing framing as a separate teaching concept per se. This also corresponds well with inter- or transdisciplinary approaches which, if well-balanced power relations are sought (Bresler 1995; Ramadier 2004), should allow nuanced perspectives on which factors to take into consideration when discussing SSIs, thereby providing a similar effect to that of making framing explicit.

### Putting the Models to the Test on the SSI of the COVID-19 Pandemic

**Fig. 5:**
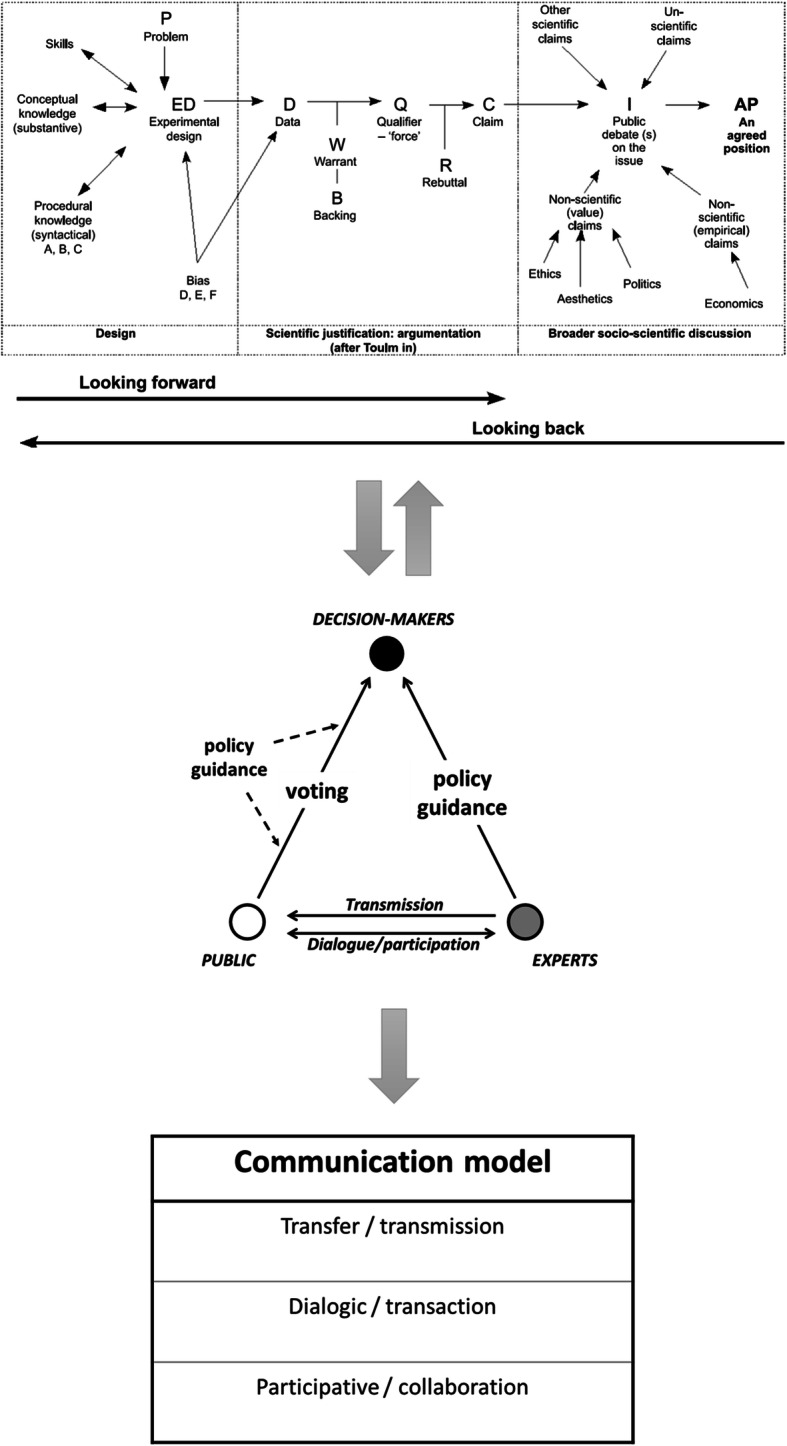
Composite processual model for conceptualising SSIs based on models from science communication and science education. Note that the lowermost element (communication model) is also included as a feature of the revised version of the middle element (expert–public–decision-maker relationships).

I seek to show how the presented models may be combined to give a composite model that sheds light on SSIs in general and the COVID-19 pandemic in particular. For a given SSI, Roberts and Gott’s model (Fig. 4) can be used to map out which questions and factors are relevant to include. Depending on the nature of the question, Feinstein’s model (Fig. 3) can then be used to discuss distribution of the right to opinion: who should be heard when making decisions and how we relate to the relevant knowledge. Based on these two factors, decisions can be made on both on an epistemic level (how do we frame the discourse and epistemic power relations?) and a concrete level (how do we organise and conduct the communication/teaching?) as to which mode of communication can/should be applied. This is visualised in the processual model shown in Fig. 5.

Under the assumption that the descriptions above of the COVID-19 pandemic in Norway and Sweden are valid,^10^ application of the composite model to the Norway-Sweden COVID issue could be as follows:

#### Upper Section

We want to discuss the different responses in the two countries, as it appears that the Swedish society has placed a comparably greater emphasis on purely epidemiological arguments than the Norwegian. In March, health authorities/agencies in the two countries both stated that there were no science-based reasons for school closure. The Norwegian government gave weight to non-scientific factors in the right-hand box, whereas the Swedish placed more weight on claims coming from the left-hand side. In Sweden, the juridical factor of freedom of movement in the right-hand side box happens to coincide with the expert advice, thus giving two separate reasons for maintaining an open society. These factors are not present in Norway. Although this model emphasises the argumentation (TAP) proceeding from scientific knowledge and practices towards social decision-making (left-to-right), argumentation also occurs to the same extent from other knowledge domains found within the right-hand side box, such as the juridical ones.

#### Middle Section

When weight is given to the epidemiological aspects, one would slide towards the right-hand side of the triangle to give the scientific authority greater weight. This resembles the Swedish situation, where greater authority is given to the experts over laypersons for public policy guidance. This expert authority is strengthened through the public custom and practice of giving an independent and prominent position to the Public Health Agency of Sweden versus the government. Thus, a factor from the right-hand side box, a constitutional one, provides the left-hand side box with a particularly strong position.^11^ Conversely, when public debate plays a more prominent role, we slide towards the left-hand side, resembling the Norwegian situation, where expert advice is balanced to a greater degree by public debate or expert-independent judgements on the part of the decision-makers. To a certain extent, this is a chicken-and-egg situation, as the choice of which factors to emphasise in the upper section is also a matter of political debate, power relations and culture. This is indicated by arrows going both ways between the upper and middle sections.

#### Lower Section

The considerations in the two first sections will have an impact on the mode of dialogue, both epistemically (who has the right of opinion, and wether a particular question is open for debate or to be considered an undisputable fact) and practically (instructional, dialogic or participatory: should we instruct/inform, discuss or engage in collective inquiry). These modes of communication take place between experts and the public, between government and the public, between government and the experts and in the classroom discourse. In the Swedish context, this is visible through the personification of expert authority in the state epidemiologist Anders Tegnell’s media appearances, providing a clearly instructional/transmission approach to the communication, followed by an appeal for certain modes of behaviour/conduct by the public. The communication by the Norwegian scientific body is likewise transmission-oriented, but the decision-making process appears to be somewhat more influenced by public debate and can be conceived as somewhat more dialogic. Although it may be perceived as paradoxical, the Swedish solution provides a larger degree of freedom for the public than the Norwegian. This is coincidental in the present case and may even change over time within the same SSI.

## Summary and Outlook

In summary, I have suggested a synthesis between didactic models from science communication and science education to produce a new composite model that can be used when seeking to understand, discuss, analyse, teach or communicate complex socio-scientific issues. The model is applied to the SSI of the COVID-19 pandemic, more specifically the contrast between the Norwegian and Swedish responses to meet the challenge. As we now observe various ways to meet the pandemic in different countries and regions, only time will show what was the most effective approach. I do not claim to have covered all facets and aspects of the pandemic in the two countries, as this is still changing on a day-to-day basis, while this article is being written. The purpose has rather been to show how the model(s) can be used to unpick and discuss the situation(s), independently of the nature and outcome of the SSI, in real time, with flexibility to pick up and discuss variable and temporally changing factors and relationships. The model also takes the multiple perspectives inherent in inter-/multi-/transdisciplinary SSIs seriously, exposing and allowing power relations to be considered, both between stakeholders in an SSI, and between knowledge domains.

As socio-scientific issue, the present pandemic is intense and past-paced and thereby different from slower-moving SSIs such as AGW, wildlife diversity, nutrition and GMO. It provides us with clear and immediate signals; you can, for instance, test yourself and receive the outcome in a matter of days of a certain behaviour, such as attending a party. Statistical data are materialising and developing in front of our eyes day by day. What is not unique with this situation is the trait of the pandemic as a liminal phase, the fact the we do not know how the world looks on the other side of COVID-19. After all, likewise when it comes to global warming, we can only imagine, or fantasise, what a modern world in climatic balance would be like (Sovacool et al. 2020; Wals and Jickling 2002). Comparing these two SSIs is thus enlightening. In terms of future perspectives, one appears to be in a fast-paced liminal phase, whereas the other is in a slow-paced liminal phase. In terms of scientific understanding, one (the ongoing pandemic) is in a state of epistemic uncertainty, whereas the other (AGW) has reached a high degree of scientific consensus. In the case where the SSI builds on knowledge beyond the ability of learners or the general public to scrutinise, we are to a greater degree left to trust-building, the ‘Lippmann approach’ (and Norris 1995), at least as long as there is a consensus within the scientific community (AGW). In the situation of the ongoing pandemic, both politicians and the public at large may seem to be expected to make their choices before the scientific jury is back with consensual results to inform decision-making. And even after this, there are still many other factors in addition to those from science that impinge on the situation. This has become evident in the contrast between neighbours Norway and Sweden.

There will always be a gap between expert knowledge and layperson knowledge (notably, an expert in one field will usually be a layperson in another). Furthermore, there will usually be a gap between the knowledge required to inform decisions and the knowledge of the layperson, neither of which are at the level of the expert. Reliance upon expert knowledge is inevitable, from both a science education and a science communication perspective. Can we, then, educate for informed decision-making, when even we as science educators should be considered laypersons, at the same time as we teach socio-scientific issues? Or are we inevitably bound to rely on second-hand inquiry on our own and our students’ parts, where teaching is reduced to trust-building or, in the extreme, opinion-making? In the words of Norris (1997, p. 256), “[a]s part of learning to live with science, students would need practice in judging the credibility of scientific experts. This practice should be based on real-world problems that currently affect their lives.” We are constantly drawn between education and opinion-making, and usually we seek a middle ground. The question remains how to balance the two. This is certainly a context-dependent question. Teacher knowledge and teaching methods will also play a significant in this balance.

Frightful and heartbreaking as it is, the present pandemic has givem reasons for why science educators and communicators would benefit from increased contact. Furthermore, it has opened a new space for science education and science communication to explore overlaps, frictions and possibilities in the intersections between the fields. The two have developed (didactic) models that can promote various modes of interplay to act as mental heuristics to bridge the two fields, thereby promoting productive cross-fertilisation and cross-pollination. though it would be overly optimistic to describe it as a direct result of the present situation, this pandemic may provide new opportunities to promote understanding of, and learning about, processes of construction of scientific knowledge, the tentative nature of scientific knowledge and its application in society. This is perhaps a major difference from the times of Lippmann and Dewey, where scientific information would often have been ready digested and carved in stone by the scientific community before reaching the classrooms and public sphere.

## Footnotes

1 Herein, the term ‘didactic’ draws on the humanistic *didaktik* and *bildung* tradition of continental Europe, referring to theory, design and art of teaching. This is not to be confused with the anglophone understanding of the term, generally avoided due to its connotations of moralising, unidirectional or patronising manners of instruction. *Didaktik* is increasingly seen also spelled with ‘c’ in the international literature, as described by, for example, Arnold (2012) in general education and Sjöström et al. (2020) in science/chemistry education. I have chosen to adhere to this latter spelling.

2 One reviewer pointed out that the Swedish approach has been subject to much debate and critique domestically and internationally and may thus be subject to revision. It is, however, not the purpose of this text to discuss the pandemic per se, however, and some of the information herein must be considered as ‘a snapshot in time’. This indeed serves to illustrate points made below in relation to our dealings with the tentative nature of scientific knowledge and the state of liminality.

3 Notably, the last part of the Swedish name for the Public Health Agency, Folkhälso*myndigheten*, translates directly as ‘authority’, and ‘the Public Health Authority’ would be a valid translation. In Norway, the public body is the *Institute* of Public Health.

4 I am indebted to one of the reviewers for pointing out these perspectives.

5 Recent alternatives to remedy this have been proposed, such as the international ‘My country talks’ project (https://www.mycountrytalks.org).

6 A visit to an online home brewing discussion forum would testify to this, that occasionally would even produce knowledge or perspectives to the benefit of academics.

7 Exceptions do exist that fall within Sadler’s (2004) definition of SSIs; see, for example, Fooladi (2013, 2020), Herranen et al. (2019) and Mata (2013).

8 The latter has, however, been suggested and is under current debate (see e.g. Chappell and Singer, 2020, and replies to this).

9 There are clearly important distinctions between ‘data’, ‘information’ and ‘knowledge’. I have deliberately kept this fluid, as an in-depth discussion would justify an article on its own. This has been discussed in depth by Roberts and colleagues (Roberts and Johnson 2015 and references therein), and amongst others in the literature on argumentation (e.g. Erduran and Jiménez-Aleixandre 2007). Still, distinctions and aspects of data versus knowledge/science-based claims can be unpicked by delving into the middle section of Fig. 4, Toulmin’s argumentation pattern, and how this bridges the two other sections.

10 The situation may, of course, change as new scientific results appear, calling for the revision of science-based recommendations for closures/lockdown. If so, the picture must be redrawn. This again serves to illustrate the dynamics between scientific knowledge in the making and society, to which the model is suited to respond.

11 Obviously, juridical aspects, like the scientific ones, are subject to an expert-public balance and such discussions may indeed be carried out for claims from any subject domain, not only science.
